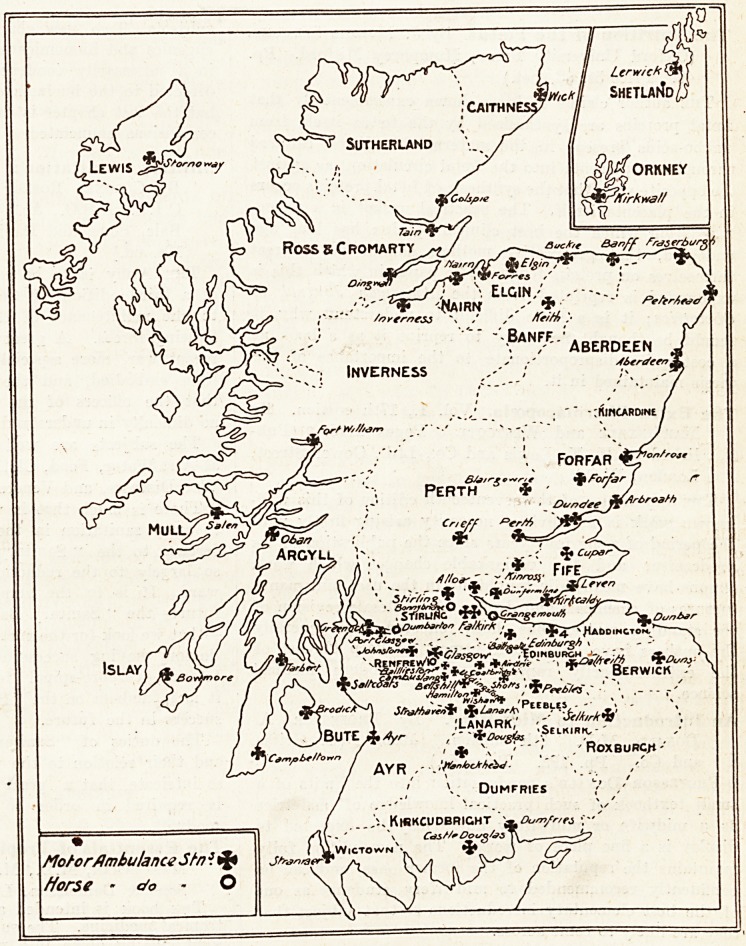# The St. Andrew's Association's Work

**Published:** 1920-09-11

**Authors:** 


### SCOTLAND'S MOTOR AMBULANCE SERVICE.

### The St. Andrew's Association's Work.

Since its inauguration in the year 1882 the St. Andrew's Ambulance Association, among its other activities, has devoted itself to the establishment of ambulance waggon services in Scotland. Before the year 1882 there was not a properly constructed ambulance waggon in Scotland. Cases of serious illness or accident were conveyed to the infirmaries and hospitals in cabs and other unsuitable vehicles. The Association began by providing two horse-drawn ambulance waggons for Glasgow, and within a few years it owned fifteen horse waggons. Three were stationed in Glasgow and the other twelve in other populous towns. The Association did everything in its power to encourage local communities to establish ambulance services, and before many years elapsed Scotland was well provided with horse-drawn waggons, over thirty of which were owned and run by the Association.

### Motors Replace Horse-drawn Vehicles.

With all their usefulness the horse-drawn vehicles had the disadvantages of lack of speed and a limited radius, and in the year 1905 the Council of the St. Andrew's Ambulance Association decided to experiment in motor traction. The horse-drawn vehicles were gradually replaced by motor waggons, and, by the year 1914, motor traction for ambulance work was used exclusively in Glasgow and the more populous centres. Prior to the war motor traction for ambulance work had been adopted in most of the larger towns in Scotland, but it was not until after the Armistice that the Association was enabled to establish a chain of motor ambulance services throughout Scotland. The relations between the Scottish Branch of the British Red Cross Society and the St. Andrew's Ambulance Association have always been of a close and cordial character. Indeed, it was due in no small measure to the Association that the Scottish Branch at the outset of its career was placed upon a solid foundation. It was, therefore, as fitting as it was generous that, when its fine fleet of motor ambulance waggons was no longer required for military purposes, the Scottish Branch of the British Red Cross Society, recognising the St. Andrew's Association as the civilian ambulance body in Scotland, placed a large number of these waggons at the Association's disposal.

### Distribution throughout Scotland.

In all, seventy-seven waggons were handed by the Scottish Branch to the Association. The hard service to which they had been put necessitated considerable repair and overhaul, for which the Scottish Branch made provision. The work of distributing these waggons was carried out by the Chairman of the Council, Colonel D. J. Mackintosh, C.B., M.V.O., LL.D., ably assisted by Mr. Wellwood R. Ferguson, the General Secretary. When the waggons were received, the Association, in addition to its headquarters in Glasgow, had twenty-four centres or local committees in Scotland. These were quite inadequate for the purpose in view, and it was found necessary to form forty-seven additional centres.

**Scotland's Motor Ambulance Waggon Service**—(*cont.*).

Scotland is now provided with a motor ambulance service extending from Wick on the north to Stranraer on the south, working in co-operation under the unified control of the St. Andrew's Ambulance Association. Separate provision has also been made for the islands. The headquarters of the St. Andrew's Ambulance Association are in Glasgow, and there are seventy-one local centres or branches, which are marked on the map accompanying this article.

A QUARTER OF A MILLION CASES TRANSPORTED.

Some figures may be of interest. In the first annual report of the St. Andrew's Association it is recorded that 372 cases had been transported in Glasgow in the previous twelve months. For the year ended May 31, 1920, the cases carried in Glasgow alone numbered 12,419, of which 5,960 were cases of accident and 6,459 of illness. In the year 1886-87 the Association's waggons in Scotland carried 846 cases. In the year 1919-20 the number was 23,891. Since the formation of the Association in 1882, 242,276 cases of accident and illness have been transported.

## Figures and Tables

**Figure f1:**